# Supplementary material for: Repurposing of promoters and enhancers during mammalian evolution
Source: Nat Commun. 2018 Oct 4;9:4066. doi: 10.1038/s41467-018-06544-z (PMC6172195; doi:10.1038/s41467-018-06544-z)
Supplement: Supplementary file 1 — Supplementary Information [file 41467_2018_6544_MOESM1_ESM.pdf]

Supplementary Information

Repurposing of promoters and enhancers during mammalian evolution

Carelli et al.

Supplementary Fig. 1

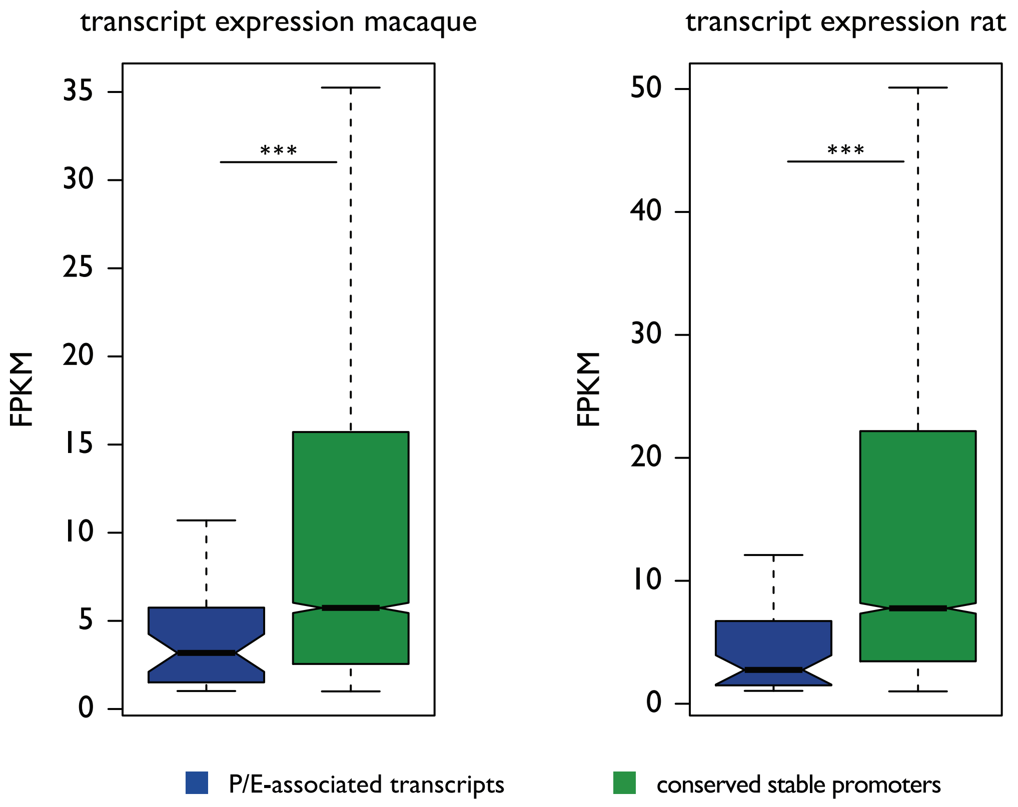

Supplementary Figure 1: Expression of macaque and rat transcripts associated to P/E elements or to promoters conserved in their sister species (human and mouse, respectively). When multiple transcripts originated from the same promoter, the sum of their individual expression level was considered. Significant differences (Mann-Whitney U test): (***) P < 0.001.

Supplementary Fig. 2

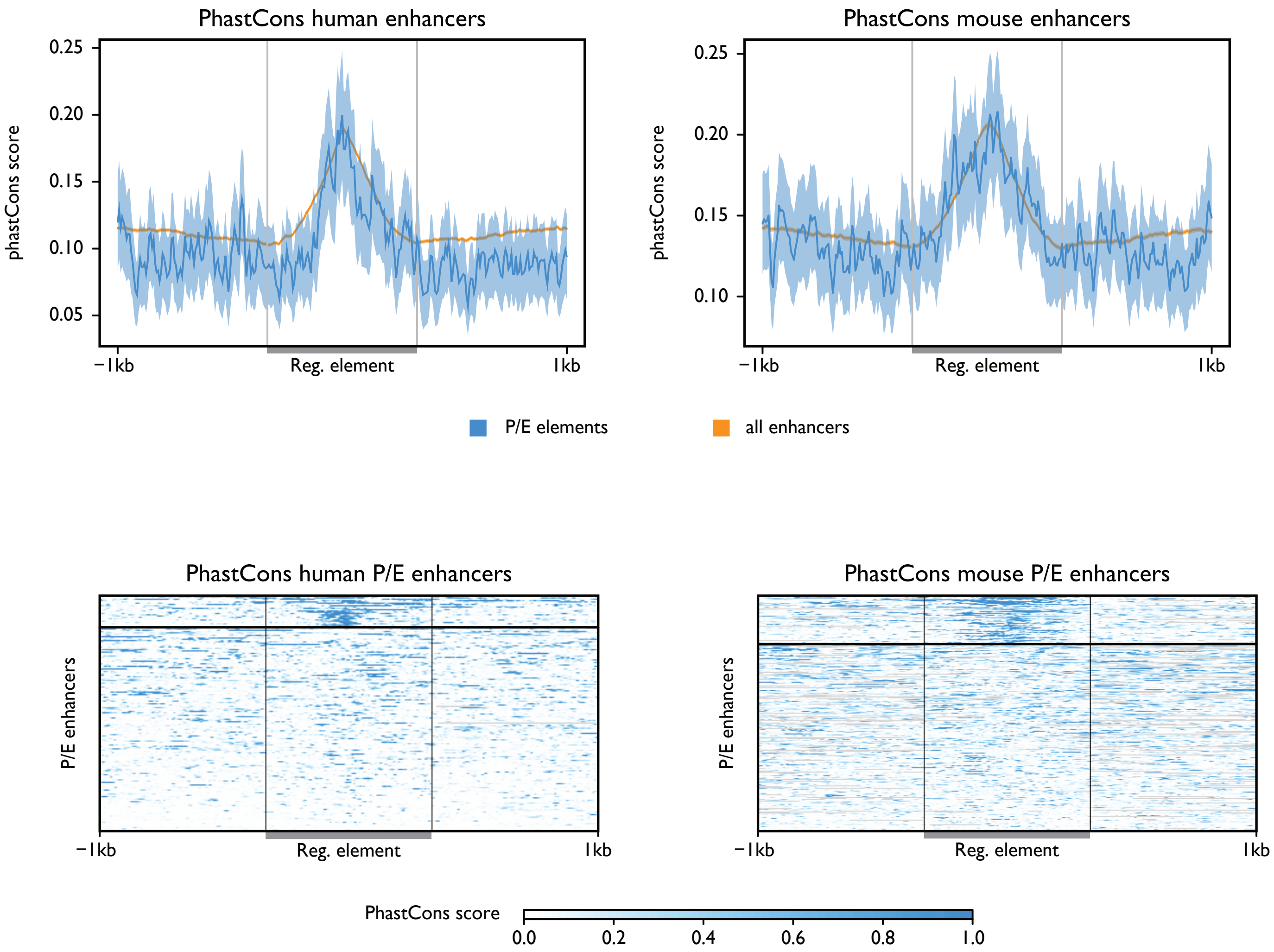

Supplementary Figure 2: (Top) PhastCons score distribution at P/E enhancers (blue) and non-P/E enhancers (orange) loci in human and mouse. PhastCons scores are displayed over the whole regulatory region (pseudoscaled to 1000 nt) and for the 1000 nt flanking regions. Continuous lines represent mean PhastCons values, shaded areas 95% confidence intervals. (Bottom) Heatmaps of PhastCons scores calculated over P/E enhancers in human and mouse. K-means clustering identifies two sets of P/E elements characterized by high or low PhastCons scores in each species.

Supplementary Fig. 3

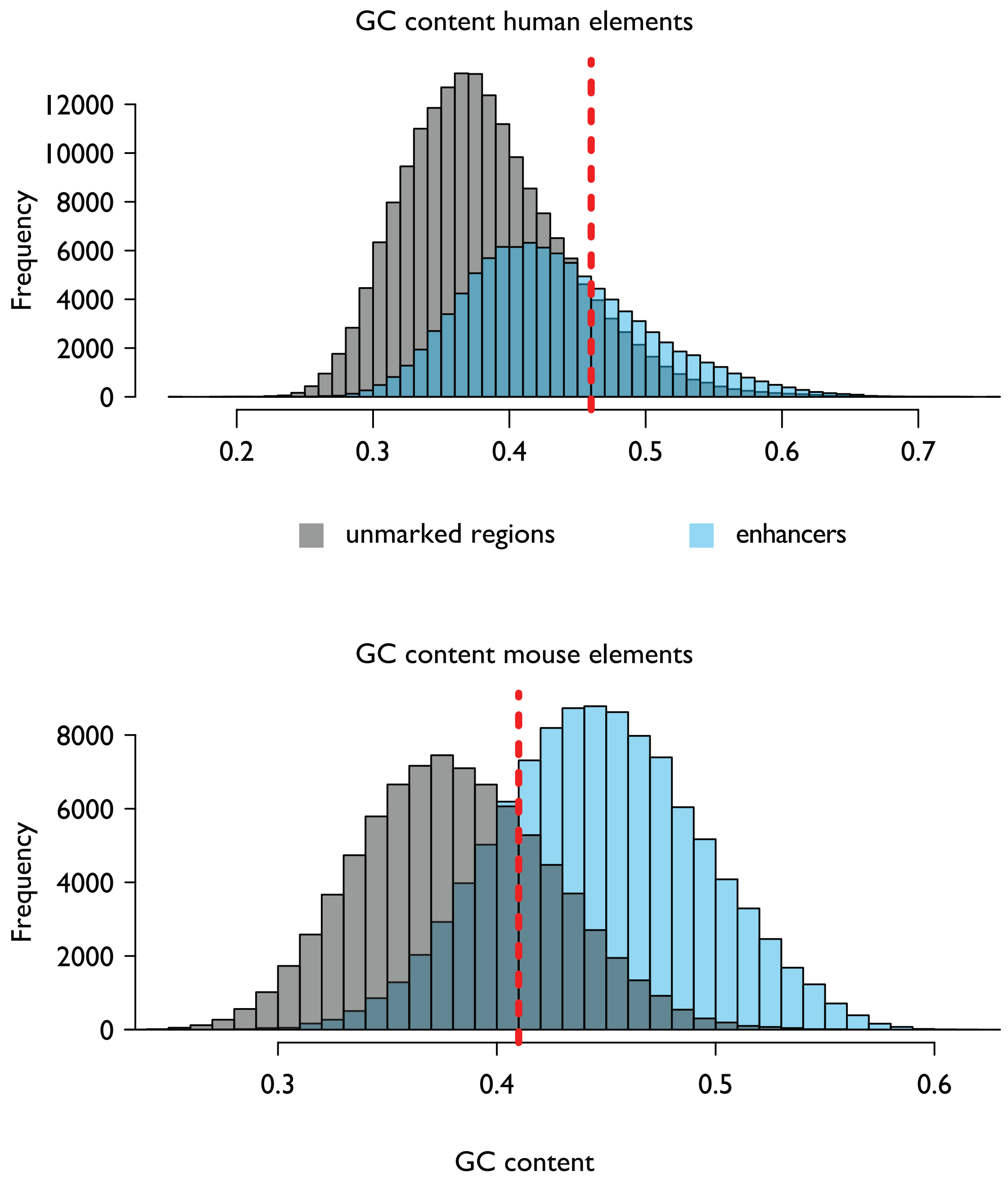

Supplementary Figure 3: GC content distribution of the whole set of inactive regions (in grey) and enhancers (in blue) in human and mouse. The dotted red lines indicate the maximum value below which we could subsample a similar number of inactive and enhancer regions with matching GC **content.**

Supplementary Fig. 4

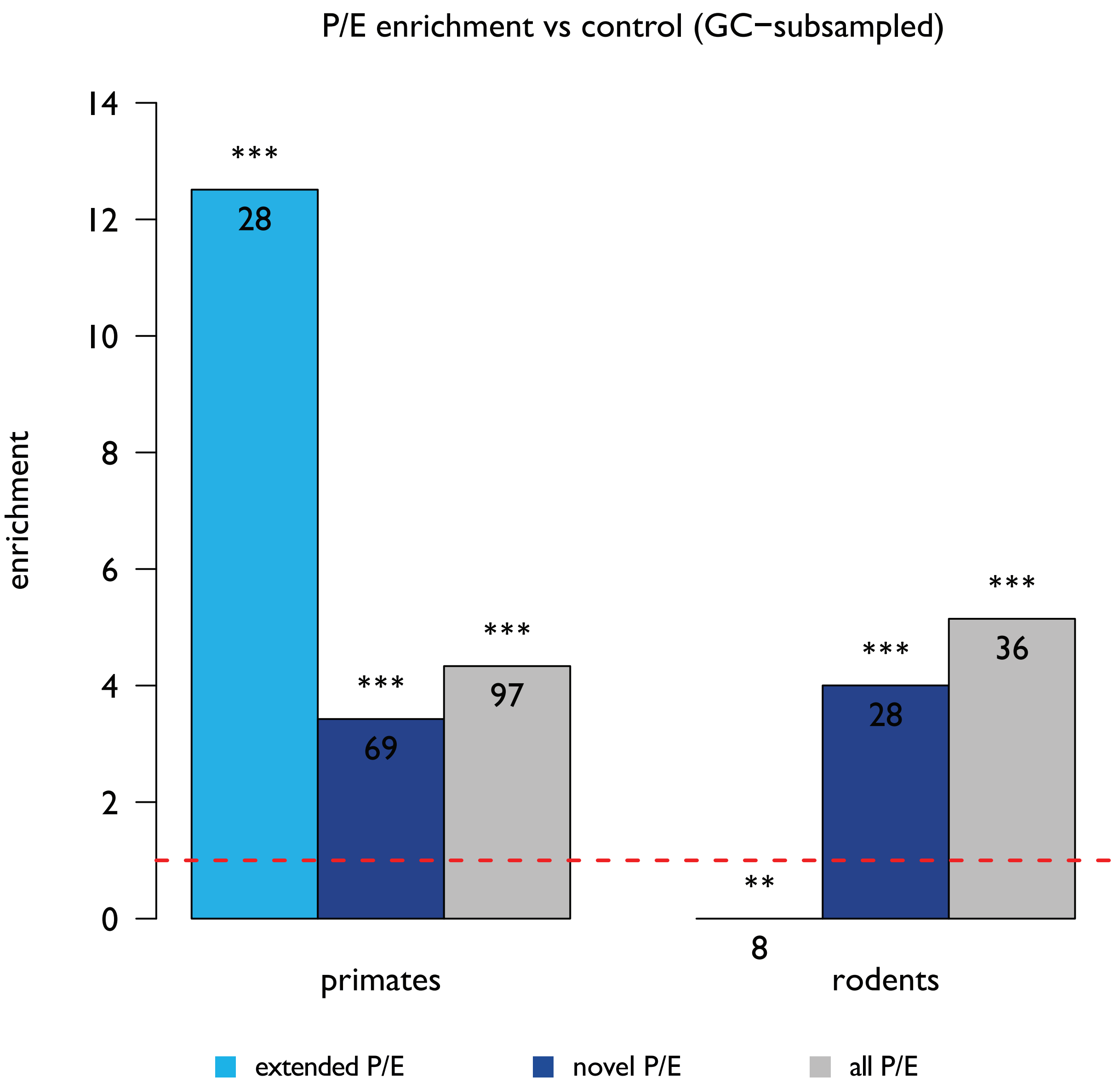

Supplementary Figure 4: Fold difference between GC-subsampled P/E elements ratio (fraction of human or mouse enhancers corresponding to promoters) and P/inactive ratio (fraction of human or mouse inactive regions corresponding to promoters). We considered only P/E elements with a GC content below 0.46 and 0.41 in human and mouse, respectively, and a random subset of inactive regions with a similar GC content distribution. The red line indicates no difference between the two ratios. Numbers in the bar plot indicate the number of P/E elements for each group. Enrichment bar not shown **for rodent extended P/E elements due to division by 0. Significant differences (Fisher's exact test with Benjamini-Hochberg correction): (***) P < 0.001; (**) P < 0.01.**

Supplementary Fig. 5

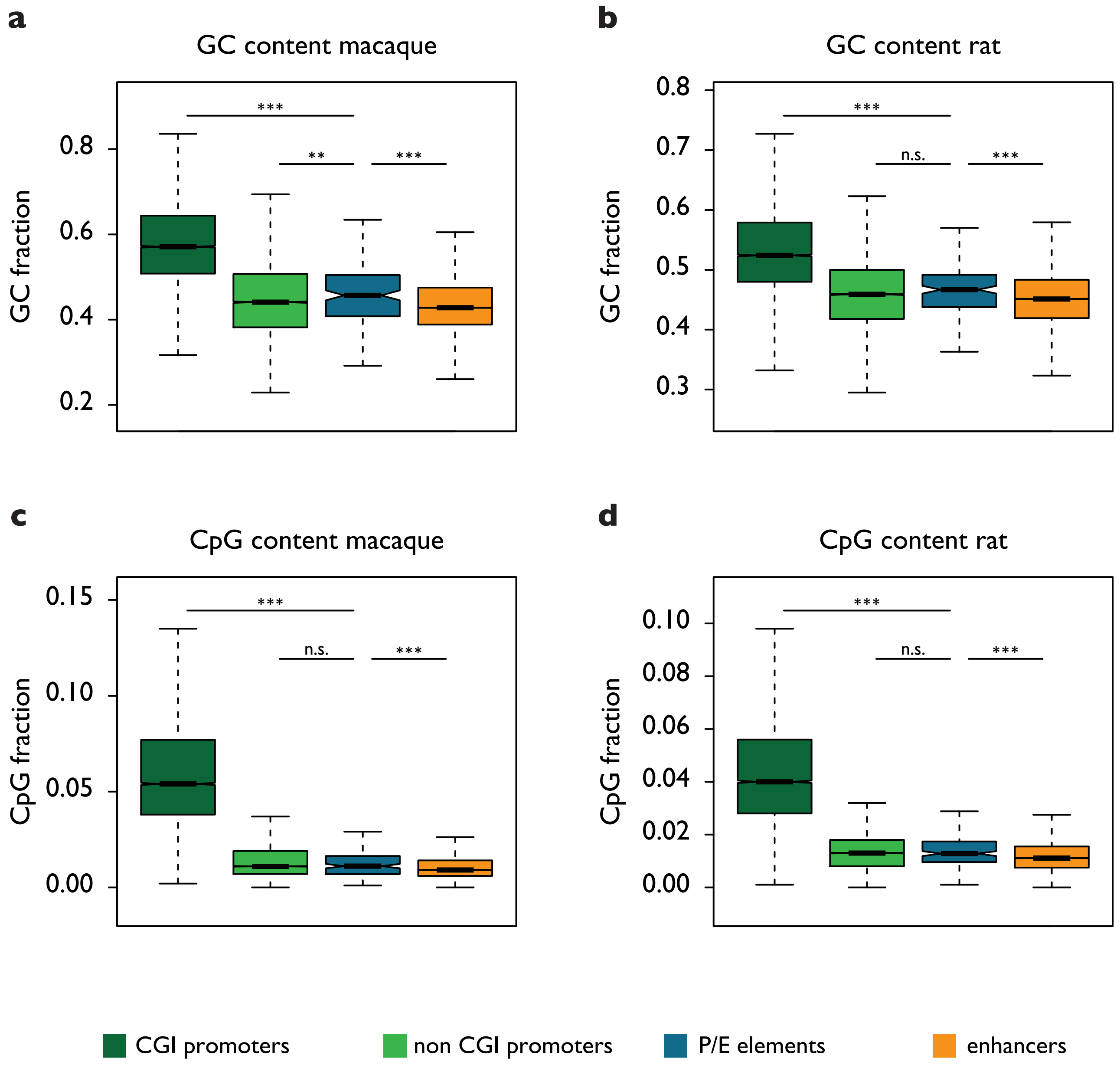

Supplementary Figure 5: Distribution of GC- and CpG-content for different classes of regulatory elements in macaque and rat. Significant differences (Mann-Whitney U test with Benjamini-Hochberg correction): (***) $P < 0.001$; (**) $P < 0.01$; (n.s.) $P \geq 0.05$.

Supplementary Fig. 6

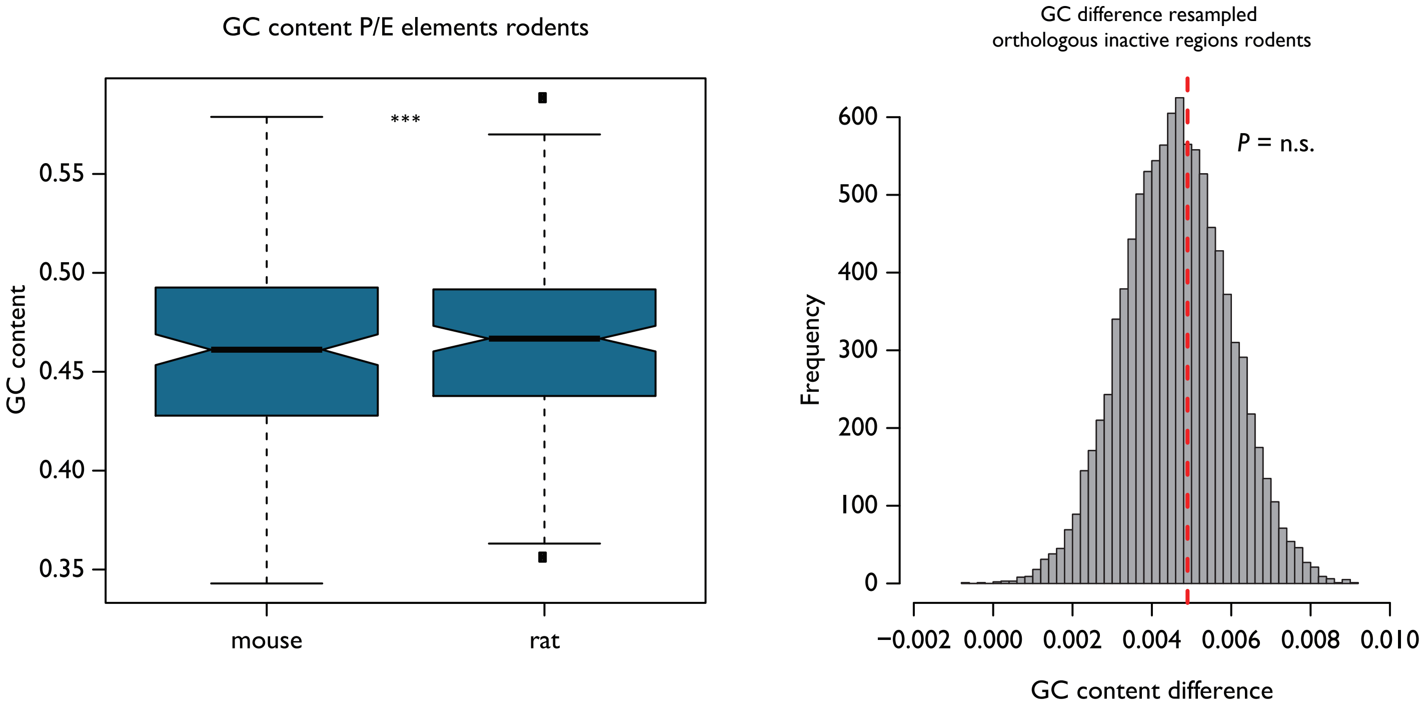

Supplementary Figure 6: Left: GC-content distribution in orthologous rodent P/E elements. Significant differences (Mann-Whitney U test): (***) P < 0.001. Right: GC content difference measured between orthologous inactive regions. The distribution was obtained by resampling 10,000 times a number of orthologous inactive regions equal to the number of P/E elements and calculating the average GC content difference. The red line indicates the mean GC content difference between orthologous rodent P/E elements. The *P*-value indicates the fraction of resampled inactive regions with an average GC content higher than that of P/E elements (n.s.: *P* > 0.05).

Supplementary Fig. 7

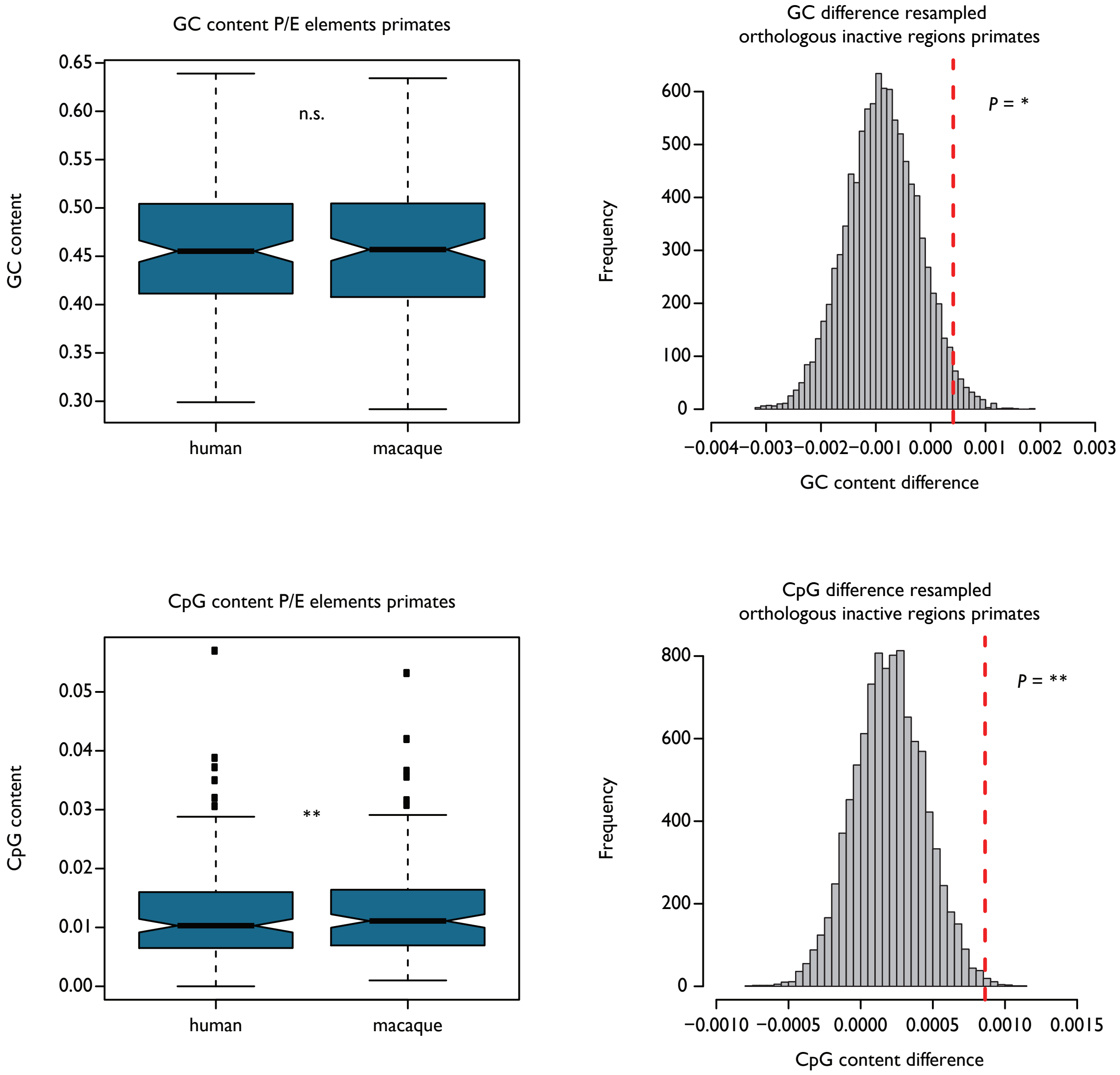

Supplementary Figure 7: Left: GC content (top) and CpG dinucleotide frequency (bottom) distribution in orthologous primate P/E elements. Significant differences (Mann-Whitney U test): (**) $P < 0.01$; (ns) $P \geq 0.05$. Right: GC content difference measured between orthologous inactiveregions. The distribution was obtained by resampling 10,000 times a number of orthologous inactive regions equal to the number of P/E elements and calculating the average GC content (top) and CpG dinucleotide frequency (bottom) difference. The red line indicates the mean GC content (top) or CpG dinucleotide frequency (bottom) difference between orthologous rodent P/E elements. The *P*-value indicates the fraction of resampled inactive regions with an average GC/CpG content higher than that of P/E elements: (**) $P < 0.01$; (*) $P < 0.05$.

Supplementary Fig. 8

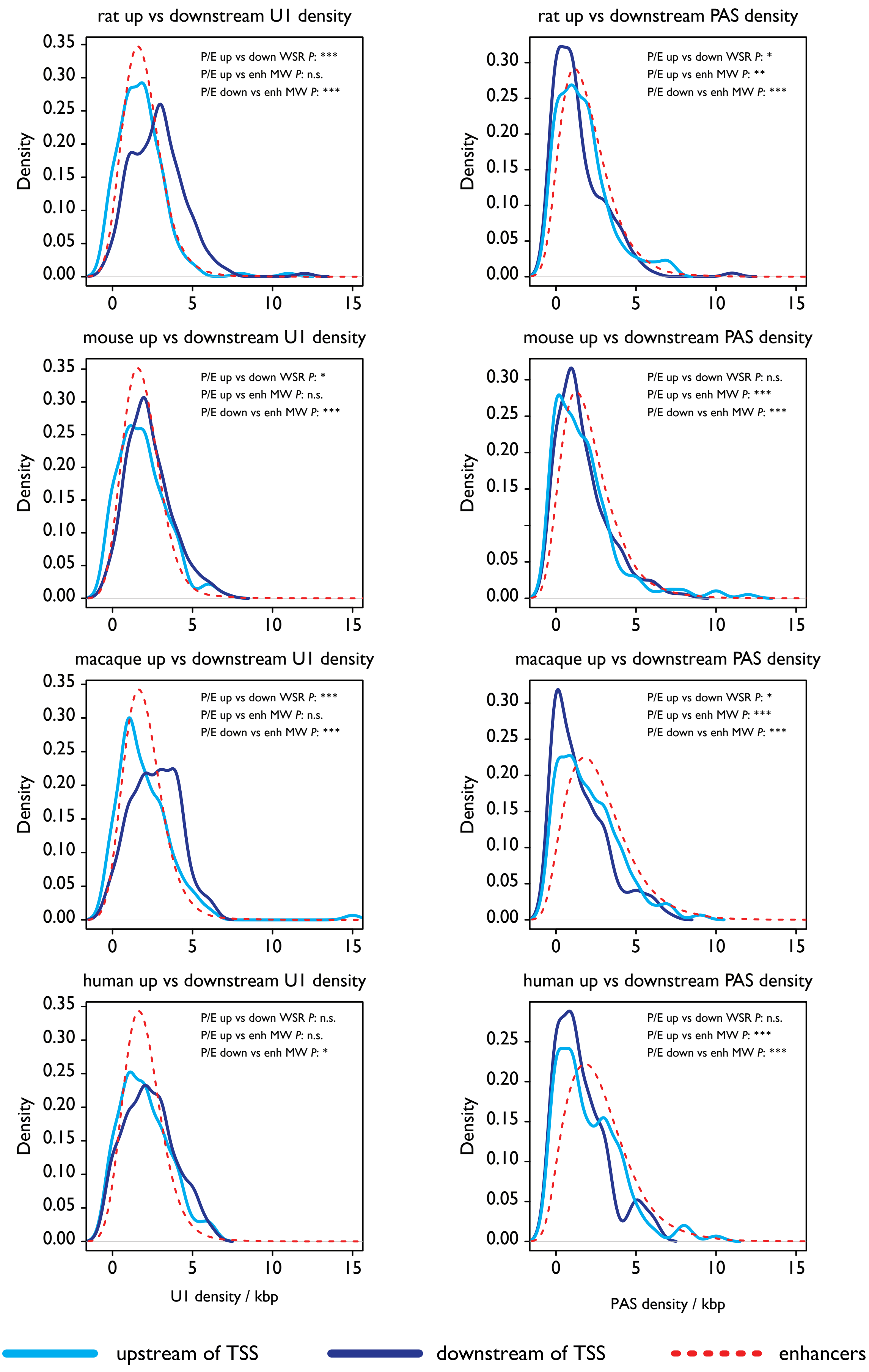

Supplementary Figure 8: Distribution of U1 and PAS motifs density per kb upstream (light blue) and downstream (dark blue) of the TSS of transcripts associated to novel P/E elements in human, macaque, mouse and rat. The dashed red lines depict the U1/PAS average density measured in the -1/+1 kbp from the TSS of CAGE-defined enhancers in human and mouse and their projected loci in macaque and rat, respectively. Statistical tests: WSR: Wilcoxon signed rank; MW: Mann-Whitney. Significant differences (after Benjamini-Hochberg correction): (***) P < 0.001; (**) P < 0.01; (*) P < 0.05; (ns) P ≥ 0.05.

Supplementary Fig. 9

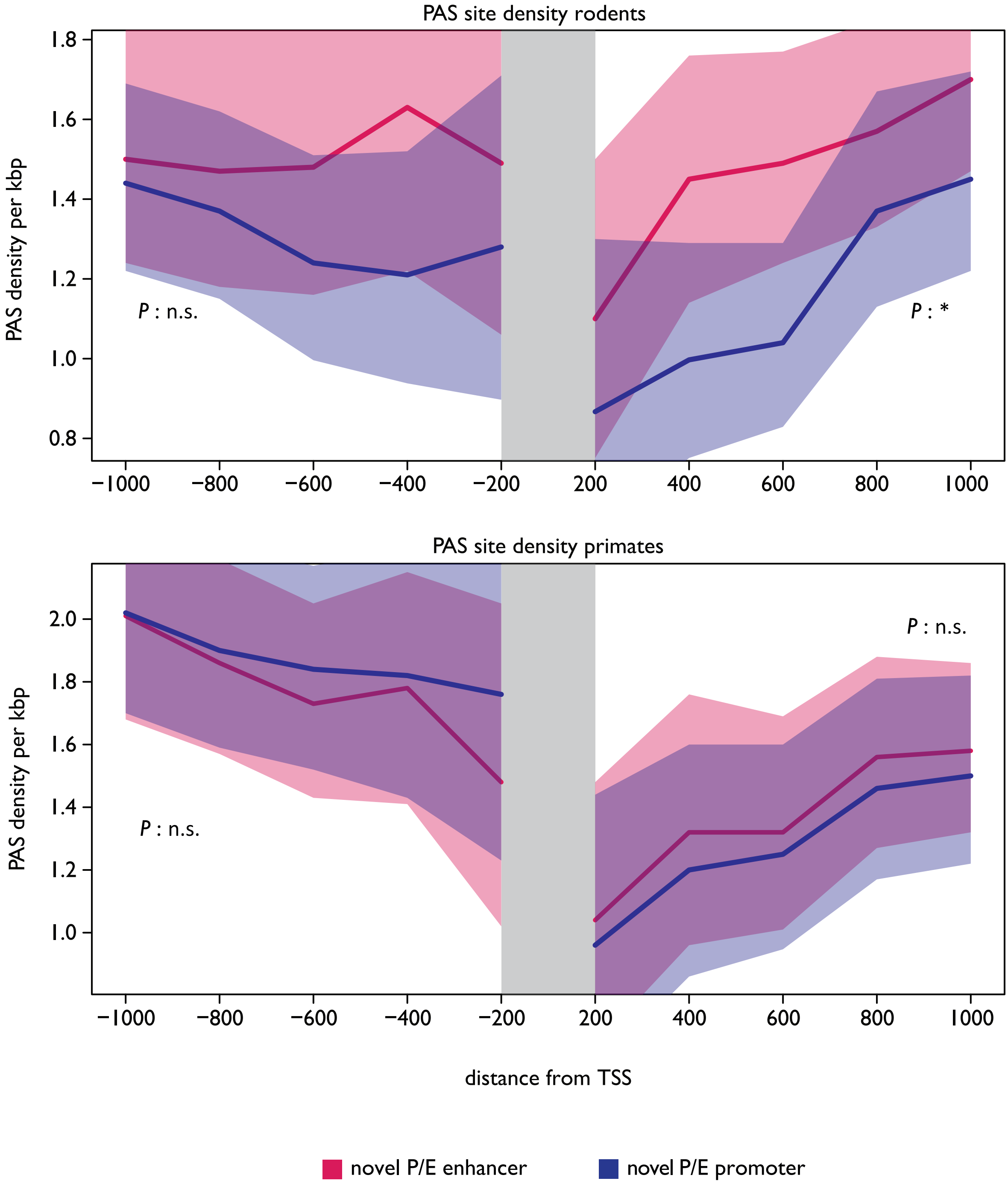

Supplementary Figure 9: Cumulative density of PAS sites up- and downstream of novel P/E-associated TSSs in rodents (up) and primates (bottom). Lines represent the mean PAS density (per kb) over 200, 400, 600, 800 and 1,000 nt long windows from the TSS, shaded areas represent 95% confidence intervals. Significant differences (Mann-Whitney U test with Benjamini-Hochberg correction): (*) $P < 0.05$; (n.s.) $P \geq 0.05$.

Supplementary Fig. 10

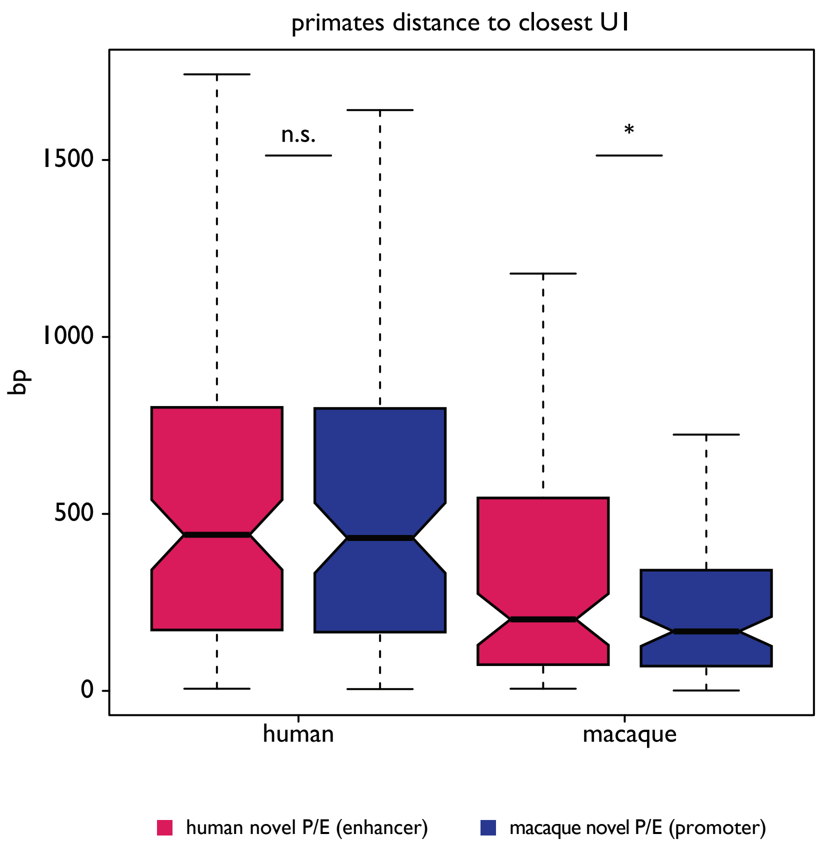

Supplementary Figure 10: Distribution of up- and downstream distances of the closest U1 site from each novel P/E-associated TSS in primates. Significant differences (One-tailed Mann-Whitney U test with Benjamini-Hochberg correction): (*) $P < 0.05$; (n.s.) $P \geq 0.05$.

Supplementary Table 1: regulatory elements conservation in outgroup species

clade	human/mouse liver enhancers	human/mouse liver promoters	enhancers aligned on all species	promoters aligned on all species	enhancers with conserved activity in all species	enhancers with no activity in outgroup	promoters with conserved activity in all species	promoters with no activity in outgroup
primate	21068	17843	19295/21068	15729/17843	2605/19295	2348/19295	3768/15729	1675/15729
rodent	28637	10837	12461/28637	4373/10837	968/12461	1932/12461	1351/4373	941/4373

Supplementary Table 2: list of RNA-seq samples used in this work

Species	Organ	Sex	Sample_name	Age	Sample_id	Read_count	Cycle_nb	RIN	RQN
Human	Brain	Male	human_brain_male1	2 years post birth	5543sTS	31347915	100	6.4	
Human	Brain	Male	human_brain_male2	39 years post birth	5574sTS	57191632	100	7.9	
Human	Brain	Male	human_brain_male3	8 years post birth	5896sTS	18323213	100		6.5
Human	Brain	Male	human_brain_male4	7 years post birth	5895sTS	18138013	100		5.2
Human	Heart	Male	human_heart_male1	2 years post birth	5839sTS	13262802	100		7.7
Human	Heart	Male	human_heart_male2	13 years post birth	5822sTS	22150161	100		7.4
Human	Heart	Female	human_heart_female1	6 months post birth	5828sTS	19795061	100		7.4
Human	Heart	Female	human_heart_female2	newborn	5843sTS	40894245	100		7.8
Human	Kidney	Male	human_kidney_male1	2 years post birth	5821sTS	28825046	100		7.6
Human	Kidney	Male	human_kidney_male2	26 years post birth	614sTS	36939502	100	7.7	
Human	Kidney	Female	human_kidney_female1	newborn	5844sTS	29328200	100		6
Human	Kidney	Female	human_kidney_female2	66 years post birth	873sTS	29452377	100	8	
Human	Liver	Male	human_liver_male1	64 years post birth	521sTS	45861554	100	7.5	
Human	Liver	Male	human_liver_male2	29 years post birth	5558sTS	17750545	100	5.4	
Human	Liver	Male	human_liver_male3	39 years post birth	5551sTS	25045771	100	8.6	
Human	Liver	Male	human_liver_male4	39 years post birth	5563sTS	31950535	100	7.2	
Macaque	Brain	Male	macaque_brain_male1	21 years post birth	5413sTS	44609464	100	NA	NA
Macaque	Brain	Male	macaque_brain_male2	9 years post birth	5382sTS	67324131	100	NA	NA
Macaque	Brain	Male	macaque_brain_male3	22 years post birth	5461sTS	32221651	100	NA	NA
Macaque	Heart	Male	macaque_heart_male1	21 years post birth	5441sTS	55159476	100	NA	NA
Macaque	Heart	Male	macaque_heart_male2	9 years post birth	5450sTS	42196163	100	NA	NA
Macaque	Heart	Male	macaque_heart_male3	22 years post birth	5401sTS	28253901	100	NA	NA
Macaque	Kidney	Male	macaque_kidney_male1	9 years post birth	5476sTS	38781442	100	NA	NA
Macaque	Kidney	Male	macaque_kidney_male2	22 years post birth	5391sTS	28915446	100	NA	NA
Macaque	Kidney	Male	macaque_kidney_male3	15 years post birth	5445sTS	43348552	100	NA	NA
Macaque	Liver	Male	macaque_liver_male1	21 years post birth	5404sTS	19319444	100	NA	NA
Macaque	Liver	Male	macaque_liver_male2	9 years post birth	5425sTS	39885337	100	NA	NA
Macaque	Liver	Male	macaque_liver_male3	22 years post birth	5395sTS	31397011	100	NA	NA
Macaque	Liver	Male	macaque_liver_male4	15 years post birth	5416sTS	15714787	100	NA	NA
Marmoset	Brain	Female	marmoset_brain_female1	11 years post birth	5791sTS	37636686	100		7.9
Marmoset	Brain	Male	marmoset_brain_male1	>15 months post birth	5801sTS	45538889	100		8
Marmoset	Brain	Male	marmoset_brain_male2	Juvenile (>5 months	5788sTS	58186050	100		8.2
Marmoset	Heart	Female	marmoset_heart_female1	4 years post birth	1492sTS	44813133	100	7.9/7.7	6.1/5.8
Marmoset	Heart	Male	marmoset_heart_male1	>15 months post birth	5814sTS	46588588	100		8.8
Marmoset	Heart	Male	marmoset_heart_male2	>15 months post birth	5795sTS	20257338	100		8.7
Marmoset	Kidney	Female	marmoset_kidney_female1	4 years post birth	1493sTS	25955512	100	6.8/3.8	5.5/1
Marmoset	Kidney	Male	marmoset_kidney_male1	Adult (age unknown)	981sTS	28586608	100	7.2	
Marmoset	Kidney	Male	marmoset_kidney_male2	>15 months post birth	5786sTS	32872586	100		8
Marmoset	Liver	Female	marmoset_liver_female1	4 years post birth	1491sTS	18238361	100	9.1/8.8	7.2/7.8
Marmoset	Liver	Male	marmoset_liver_male1	Adult (age unknown)	983sTS	105720755	100	6.3	
Marmoset	Liver	Male	marmoset_liver_male2	>15 months post birth	5787sTS	40047815	100		10
Mouse	Brain	Male	mouse_brain_male1	9 weeks post birth	1944sTS	20425066	100		7.2
Mouse	Brain	Male	mouse_brain_male2	9 weeks post birth	1954sTS	33768896	100		7.5
Mouse	Brain	Male	mouse_brain_male3	4 weeks post birth	1892sTS	21275419	100		7.4
Mouse	Brain	Male	mouse_brain_male4	4 weeks post birth	1896sTS	19032489	100		7.1
Mouse	Heart	Male	mouse_heart_male1	9 weeks post birth	2663sTS	58066902	100		6.8
Mouse	Heart	Male	mouse_heart_male2	9 weeks post birth	1956sTS	41056894	100		6.8

Mouse	Heart	Male	mouse_heart_male3	4 weeks post birth	2659sTS	20958151	100	7.4
Mouse	Heart	Male	mouse_heart_male4	4 weeks post birth	2661sTS	35465489	100	7.4
Mouse	Kidney	Male	mouse_kidney_male1	9 weeks post birth	2664sTS	74586163	100	7.2
Mouse	Kidney	Male	mouse_kidney_male2	9 weeks post birth	1957sTS	9750709	100	7.6
Mouse	Kidney	Male	mouse_kidney_male3	4 weeks post birth	2660sTS	32156633	100	8.2
Mouse	Kidney	Male	mouse_kidney_male4	4 weeks post birth	2662sTS	27464452	100	9.8
Mouse	Liver	Male	mouse_liver_male1	9 weeks post birth	1946sTS	29472470	100	7.9
Mouse	Liver	Male	mouse_liver_male2	9 weeks post birth	1958sTS	36683243	100	7.6
Mouse	Liver	Male	mouse_liver_male3	4 weeks post birth	1894sTS	21747013	100	8.1
Mouse	Liver	Male	mouse_liver_male4	4 weeks post birth	1898sTSm	32126490	100	7.9
Rat	Brain	Male	rat_brain_male1	16 weeks post birth	2356sTS	18669353	100	7.6
Rat	Brain	Male	rat_brain_male2	16 weeks post birth	2367sTS	47743419	100	8
Rat	Brain	Male	rat_brain_male3	6 weeks post birth	1875sTS	31815993	100	7.1
Rat	Brain	Male	rat_brain_male4	6 weeks post birth	1971sTS	37935977	100	7.7
Rat	Heart	Male	rat_heart_male1	16 weeks post birth	2747sTS	27585164	100	6.6
Rat	Heart	Male	rat_heart_male2	16 weeks post birth	2749sTS	23007264	100	7.6
Rat	Heart	Male	rat_heart_male3	6 weeks post birth	2742sTS	27360682	100	7.9
Rat	Heart	Male	rat_heart_male4	6 weeks post birth	2751sTS	26404675	100	8.6
Rat	Kidney	Male	rat_kidney_male1	16 weeks post birth	2748sTS	33451376	100	7.5
Rat	Kidney	Male	rat_kidney_male2	16 weeks post birth	2750sTS	9750709	100	7.4
Rat	Kidney	Male	rat_kidney_male3	6 weeks post birth	2743sTS	27514276	100	6.3
Rat	Kidney	Male	rat_kidney_male4	6 weeks post birth	2856sTS	27570406	100	6.8
Rat	Liver	Male	rat_liver_male1	16 weeks post birth	2359sTS	29338454	100	7.8
Rat	Liver	Male	rat_liver_male2	16 weeks post birth	2370sTS	39698149	100	7.9
Rat	Liver	Male	rat_liver_male3	6 weeks post birth	1969sTS	30730476	100	8.1
Rat	Liver	Male	rat_liver_male4	6 weeks post birth	1973sTS	40753892	100	8
Rabbit	Brain	Male	rabbit_brain_male1	1 year post birth	3017sTS	33359809	100	6.7
Rabbit	Brain	Male	rabbit_brain_male2	6 months post birth	3025sTS	34477933	100	6.9
Rabbit	Brain	Female	rabbit_brain_female1	6 months post birth	3011sTS	30110598	100	7.3
Rabbit	Brain	Female	rabbit_brain_female2	6 months post birth	3021sTS	58218690	100	6.6
Rabbit	Heart	Male	rabbit_heart_male1	1 year post birth	3019sTS	41092592	100	7.1
Rabbit	Heart	Male	rabbit_heart_male2	6 months post birth	3027sTS	35150735	100	7.2
Rabbit	Heart	Female	rabbit_heart_female1	9 months post birth	3085sTS	28187893	100	7.1
Rabbit	Kidney	Male	rabbit_kidney_male1	6 months post birth	3047sTS	30756002	100	7.6
Rabbit	Kidney	Male	rabbit_kidney_male2	6 months post birth	3028sTS	22647780	100	7.6
Rabbit	Kidney	Female	rabbit_kidney_female1	6 months post birth	3014sTS	58780259	100	7.4
Rabbit	Kidney	Female	rabbit_kidney_female2	6 months post birth	3023sTS	36651975	100	7.8
Rabbit	Liver	Male	rabbit_liver_male1	1 year post birth	3079sTS	31357550	100	8.1
Rabbit	Liver	Male	rabbit_liver_male2	6 months post birth	3029sTS	28637392	100	7.6
Rabbit	Liver	Female	rabbit_liver_female1	6 months post birth	3015sTS	24614575	100	7.5
Rabbit	Liver	Female	rabbit_liver_female2	6 months post birth	3081sTS	22898659	100	8

Published in Marin et al., Genome Research, 2017
