# Supplementary material for: Repurposing of promoters and enhancers during mammalian evolution
Source: Nat Commun. 2018 Oct 4;9:4066. doi: 10.1038/s41467-018-06544-z (PMC6172195; doi:10.1038/s41467-018-06544-z)
Supplement: Supplementary file 3 — Description of Additional Supplementary Files [file 41467_2018_6544_MOESM3_ESM.pdf]

Description of Additional Supplementary Files

File Name: Supplementary Data 1

Description: transcripts assembled using RNA-seq data for each species. Transcripts were first assembled using StringTie by pooling all replicates from each organ. Organ-specific annotation was then merged using Cuffmerge. Annotations are in BED12 format.

File Name: Supplementary Data 2

Description: expression levels in FPKM for all assembled transcripts of each species as measured by Cuffnorm.

File Name: Supplementary Data 3

Description: coordinates (in BED3 format) of annotated promoter regions from each species, as described in the Methods section.

File Name: Supplementary Data 4

Description: coordinates (in BED4 format) of annotated enhancer regions from human and mouse, as described in the Methods section.

File Name: Supplementary Data 5

Description: table including orthologous regions of human and mouse enhancers in other primate (macaque and marmoset) or glires (rat and rabbit) genomes, as described in the Methods section. Human and mouse enhancers not aligned to any species are not shown.

File Name: Supplementary Data 6

Description: coordinates (in BED4+1 format) of P/E elements annotated in human, macaque, mouse and rat. The fifth column specifies whether the P/E element is “novel” or “extended”.

File Name: Supplementary Data 7

Description: Enrichment of POLII motifs (from JASPAR) for different sets of regulatory elements in human, macaque, mouse and rat. Enrichment calculated using AME as described in the Methods section.

File Name: Supplementary Data 8

Description: List of published ChIP-seq and DNase-seq files used in the present study.

File Name: Supplementary Data 9

Description: Full code used to download and process all data presented in this study.
